# Efficacy and safety of focal pulsed-field ablation for ventricular arrhythmias: two-centre experience

**DOI:** 10.1093/europace/euae192

**Published:** 2024-07-11

**Authors:** Petr Peichl, Alan Bulava, Dan Wichterle, Filip Schlosser, Predrag Stojadinović, Eva Borišincová, Peter Štiavnický, Jana Hašková, Josef Kautzner

**Affiliations:** Department of Cardiology, IKEM, Vídeňská 1958/9, Praha 4, Prague 140 21, Czechia; České Budějovice Hospital and Faculty of Health and Social Sciences, University of South Bohemia in České Budějovice, České Budějovice, Czechia; Department of Cardiology, IKEM, Vídeňská 1958/9, Praha 4, Prague 140 21, Czechia; Department of Cardiology, IKEM, Vídeňská 1958/9, Praha 4, Prague 140 21, Czechia; Department of Cardiology, IKEM, Vídeňská 1958/9, Praha 4, Prague 140 21, Czechia; Department of Cardiology, IKEM, Vídeňská 1958/9, Praha 4, Prague 140 21, Czechia; Department of Cardiology, IKEM, Vídeňská 1958/9, Praha 4, Prague 140 21, Czechia; Department of Cardiology, IKEM, Vídeňská 1958/9, Praha 4, Prague 140 21, Czechia; Department of Cardiology, IKEM, Vídeňská 1958/9, Praha 4, Prague 140 21, Czechia

**Keywords:** Catheter ablation, Ventricular premature complexes, Ventricular tachycardia, Pulsed-field energy

## Abstract

**Aims:**

A pulsed electric field (PF) energy source is a novel potential option for catheter ablation of ventricular arrhythmias (VAs) as it can create deeper lesions, particularly in scarred tissue. However, very limited data exist on its efficacy and safety. This prospective observational study reports the initial experience with VA ablation using focal PF.

**Methods and results:**

The study population consisted of 44 patients (16 women, aged 61 ± 14years) with either frequent ventricular premature complexes (VPCs, 48%) or scar-related ventricular tachycardia (VT, 52%). Ablation was performed using an irrigated 4 mm tip catheter and a commercially available PF generator. On average, 16 ± 15 PF applications (25 A) were delivered per patient. Acute success was achieved in 84% of patients as assessed by elimination of VPC or reaching non-inducibility of VT. In three cases (7%), a transient conduction system block was observed during PF applications remotely from the septum. Root analysis revealed that this event was caused by current leakage from the proximal shaft electrodes in contact with the basal interventricular septum. Acute elimination of VPC was achieved in 81% patients and non-inducibility of VT in 83% patients. At the 3-month follow-up, persistent suppression of the VPC was confirmed on Holter monitoring in 81% patients. In the VT group, the mean follow-up was 116 ± 75 days and a total of 52% patients remained free of any VA.

**Conclusion:**

Pulsed electric field catheter ablation of a broad spectrum of VA is feasible with acute high efficacy; however, the short-term follow-up is less satisfactory for patients with scar-related VT.

## Graphical Abstract

**Graphical Abstract:**
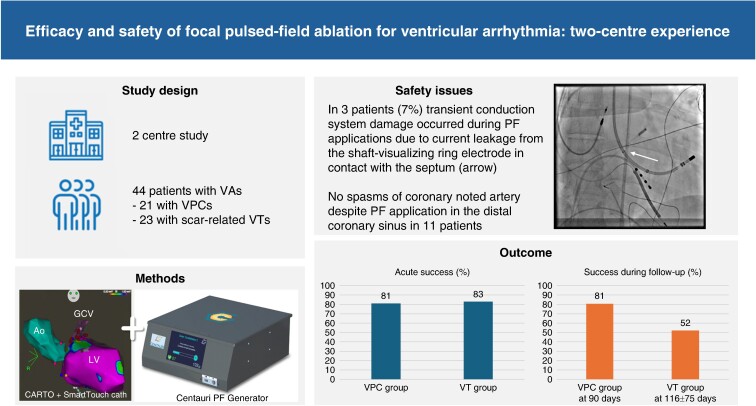

**What’s new?**

- Ablation of ventricular arrhythmia using pulsed electric field (PF) delivered from a solid-tip 4 mm catheter is feasible with a high acute efficacy; however, despite favourable acute results, the recurrences are common in the ventricular tachycardia group and acute non-inducibility may not be the optimal endpoint.
- Compared to radiofrequency energy, focal PF ablation within the great cardiac vein was not limited by a high impedance or poor catheter-tip cooling and was not associated with coronary artery spasm.
- Unexpected conduction system block was observed during retrograde catheter ablation in the left ventricle due to current leakage from the proximal, shaft-visualizing electrodes of the ablation catheter.

## Introduction

Catheter ablation (CA) is a well-established treatment of ventricular arrhythmias (VAs).^1^ In patients with frequent ventricular premature complexes (VPCs), eliminating ectopic focus may improve symptoms or lead to normalization of left ventricular (LV) ejection fraction in case of arrhythmia-induced cardiomyopathy. In patients with sustained scar-related ventricular tachycardias (VTs), CA decreases the number of therapies from the implantable cardioverter-defibrillator (ICD) and VA-related hospitalizations^2^ and may improve prognosis.^3^ Until now, radiofrequency (RF) current has been the primary energy source employed for these procedures. However, the creation of deep lesions by RF ablation might be compromised in scar regions and associated with the risk of tissue overheating and steam pop.^4^ A pulsed electric field (PF) is a novel energy source that enables fast creation of non-thermal lesions and may overcome some of the limitations of RF energy.

Currently, multiple systems allowing VA ablation by PF are in the phase of preclinical or early clinical evaluation.^5–7^ The CENTAURI system (CardioFocus) is a novel PF generator that enables PF ablation using different commercially available catheters. The system delivers a biphasic, monopolar pulsed field at three selectable energy settings (19, 22, and 25 A) that is synchronized to the R-wave. Its safety and efficacy were evaluated for ablation of atrial fibrillation.^8^ Anecdotally, this generator has been used for VA ablation, but so far, data on efficacy and safety are limited to the case reports and small case series.^9–11^ Our study aimed to analyse the safety and efficacy of VA ablation using focal PF delivered by the CENTAURI generator coupled with a contact force-sensing ablation catheter and a 3D electroanatomical mapping system in a broad population of patients with frequent VPC or scar-related VT.

## Methods

### Study population and study design

This two-centre study included consecutive patients who underwent CA for VA between May 2023 and January 2024 using the CENTAURI generator. Initially, patients with VPC from the right ventricular (RV) outflow tract were included to assess the feasibility of PF ablation. However, after seven uneventful cases, the inclusion criteria expanded to patients with other VA that failed previous RF ablation (both during the same or the previous procedure). The patient was considered non-eligible for PF ablation if the VA originated from the vicinity of the AV node or proximal conduction system. All patients signed informed consent with the procedure. The institutional Ethics Committee approved the study.

### Catheter ablation procedure

The procedures were performed under conscious sedation with fentanyl and midazolam, or on propofol. After obtaining vascular access, unfractionated heparin was administered as an initial bolus, and further doses were adjusted to maintain the activated clotting time between 300 and 350 s. The LV was accessed either transseptally or retrogradely, depending on the VA origin, the actual INR level, the presence of peripheral arterial disease, and/or mechanical valve prosthesis. Procedures were navigated using a 3D electroanatomic mapping system (CARTO 3, Biosense Webster) and guided by intracardiac echocardiography (ICE; AcuNav, Siemens Medical Solutions). For mapping and ablation, a 3.5 mm irrigated-tip catheter (ThermoCool SmartTouch™, Biosense Webster) was used. Radiofrequency energy was delivered by SMARTABLATE (Biosense Webster) set to an output of 30–40 W for up to 60 s and titrated to reach an impedance drop of 10–15 Ohms. When PF was used, 25 A applications were delivered using the CENTAURI generator and repeated at each target site up to three times to maximize the lesion size. In the VPC group, these additional applications were delivered, only when the ectopy was eliminated by the initial pulse. In the VT group, repeated applications were delivered to each targeted site and inducibility was assessed only after delivering the planned lesion set.

For patients with frequent VPC, activation mapping was used as the primary mapping strategy, complemented by pacemapping. Catheter ablation targeted the site of the earliest activity during VPC. The procedure was considered acutely successful if the clinical VPC was eliminated despite the isoproterenol challenge.

For patients with scar-related VT, mapping and ablation strategy was described previously.^12^ Briefly, one quadripolar catheter was inserted into the right ventricle for pacing. At baseline, programmed ventricular stimulation from the RV apex was performed at two drive trains (600 and 400 ms) and up to three extrastimuli. Substrate mapping was performed primarily during spontaneous rhythm or RV pacing using an integrated approach. Bipolar voltage maps (the lower threshold of 0.5 mV) were constructed and fragmented or late potentials were tagged. Zones of slow conduction were identified by the stimulus-to-QRS onset interval longer than 40 ms. The paced QRS morphology during sinus rhythm was used to match the exit sites of induced VTs. Activation and entrainment mapping were used for well-tolerated VT. The goal of subsequent CA was to abolish all abnormal signals or late potentials, often reaching isolation of the segment of the scar with no capture. In the case of tolerated VT, CA aimed to terminate the arrhythmia. The procedure was considered acutely successful when non-inducibility of any VT was achieved.

Whenever PF was applied within the great cardiac vein, coronary angiography was performed before and after the PF energy delivery to rule out spasms of the coronary arteries. The distance between the tip of ablation catheter and the coronary artery was measured (contour to contour) at the PF application site. No nitrates were applied prophylactically prior to PF applications.

In one of the centres, peripheral venous blood samples for the assessment of the serum levels of high-sensitivity troponin T (hsTnT) were obtained the next day (usually 18–24 h after the CA).

### Clinical follow-up

Following CA, patients were evaluated in the outpatient clinic in 3-month intervals. Those with frequent VPC underwent 24-h Holter monitoring and CA were considered successful if the clinical VPC burden was significantly decreased (<20% of the pre-ablation level). Patients with scar-related VT were seen regularly in 3- or 6-month intervals, and the recurrence of VT was assessed by clinical history and ICD interrogation.

### Statistical analysis

Continuous variables were expressed as means with standard deviations and compared with Student’s *t*-test. Categorical variables were expressed as percentages and compared by Fisher’s exact test. A *P* < 0.05 was considered significant.

## Results

The population consists of 44 patients recruited in the two centres. A total of 57% of patients had previously failed RF ablation procedure(s) for VA. Twenty-one (48%) patients had frequent VPC with a mean burden of 27 ± 12% on a 24-h Holter monitoring. Twenty-three (52%) patients had scar-related VT. Baseline characteristics are displayed in *Table 1*.

**Table 1:** Baseline characteristics

	All patients	Patients with VPCs	Patients with VT	*P-*value
	*n* = 44	*n* = 21	*n* = 23	
Male sex (%)	64	47	78	0.06
Age (years)	61 ± 14	56 ± 13	63 ± 15	0.18
Body mass index (kg/m^2^)	31 ± 5	30 ± 5	31 ± 5	0.62
Diabetes mellitus (%)	23	19	26	0.72
Arterial hypertension (%)	73	62	83	0.18
Structural heart disease (%)	55	4	96	<0.001
Mean LV ejection fraction (%)	45 ± 16	58 ± 9	33 ± 10	<0.001
Previous unsuccessful RF ablation (%)	57	38	74	<0.01

LV, left ventricular; RF, radio frequency ablation; VPC, ventricular premature contraction; VT, ventricular tachycardia.

In the VPC group, ectopy originated from the LV outflow tract, RV outflow tract, posteromedial LV papillary muscle, and posterobasal LV region in 52, 33, 10, and 5%, respectively. In the VT group, the ablation was performed in the lateral LV, LV outflow tract/great cardiac vein, anterior LV wall, lateral RV wall, inferior LV wall, LV papillary muscle, and RV outflow tract in 35, 26, 13, 9, 9, 4, and 4%, respectively.

The mean procedural duration was 113 ± 46 min, and the fluoroscopy time reached 6.9 ± 4.3 min with a radiation dose of 8521 ± 12 393 mGy/cm2 (*Table 2*). On average, 16 ± 15 PF applications (25 A) were delivered per patient. The PFs were well tolerated in analgosedation, and no generalized muscle contractions that would affect the alignment of electroanatomical maps were observed. Importantly, PF deliveries did not induce sustained VT or ventricular fibrillation in any of the patients. In nine patients (20%), RF delivery was attempted and failed prior PF applications (2 ± 7 applications per patient).

**Table 2:** Procedural characteristics and outcome

	All patients	Patients with VPCs	Patients with VT	*P-*value
	*n* = 44	*n* = 21	*n* = 23	
Procedural duration (min)	113 ± 46	84 ± 41	139 ± 33	<0.001
Fluoroscopy time (min)	6.9 ± 4.3	7 ± 4	7 ± 4	0.77
Fluoroscopy dose (mGy/cm^2^)	8521 ± 12 393	8226 ± 10 657	8791 ± 14 030	0.88
PF applications per patient (*n*)	16 ± 15	7 ± 4	24 ± 16	<0.001
Acute success (%)	82	81	83	1.0
Absence of recurrences during follow-up (%)	66	81	52	0.06

PF, pulsed field; VPC, ventricular premature complex; VT, ventricular tachycardia.

### Pulsed electric field ablation in the great cardiac vein

**Figure 1:**
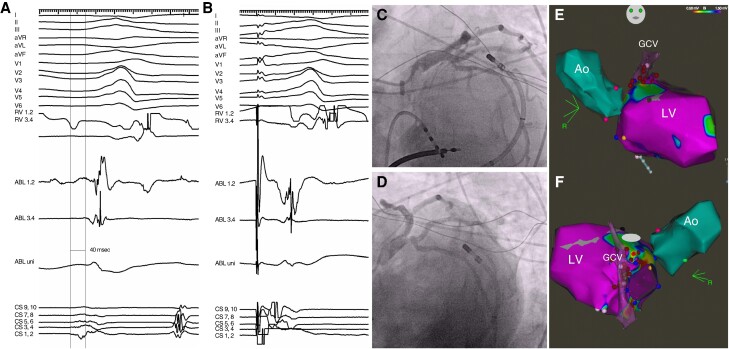
An illustrative case of ablation in the great cardiac vein in a patient with non-ischaemic cardiomyopathy and scar-related VT from the LV summit. (*A*) shows prematurity (−40 ms) and fragmentation during the VPC in the decapolar catheter positioned close to the substrate in the great cardiac vein (CS 3.4). Note relatively late activation in the ablation catheter positioned on the endocardium of the LV outflow tract. (*B*) shows the corresponding pace map with a long stimulus-to-QRS delay. (*C*) depicts angiography of the left coronary artery position prior to ablation. No spasm (*D*) was noted after four PF applications in the great cardiac vein. (*E* and *F*) display electroanatomical maps in anteroposterior (*E*) and modified cranial view (*F*). Ao, aorta; ABL, electrograms from ablation catheter; CS, coronary sinus; GCS, great cardiac vein; LV, left ventricular; PF, pulsed field; RV, right ventricle; VPC, ventricular premature complex.

In 11 cases (8 and 3 in the VPC and VT groups, respectively), PF energy was applied in the great cardiac vein up to 2 mm from the coronary artery (mean distance of 5 ± 2 mm). No electrocardiogram changes attributable to ischaemia were noted after the PF applications, and subsequent coronary angiography did not reveal any abnormality/spasm in any of these patients (*Figure 1*). In 7 of 11 patients (63%), PF ablation led to acute suppression of VA. The mean prematurity during VPC/VT in patients was higher in those with acutely successful ablation compared to those where no acute effect was seen (31 ± 8 ms vs. 18 ± 5 ms, *P* = 0.08).

### Conduction system block during pulsed electric field applications

**Figure 2:**
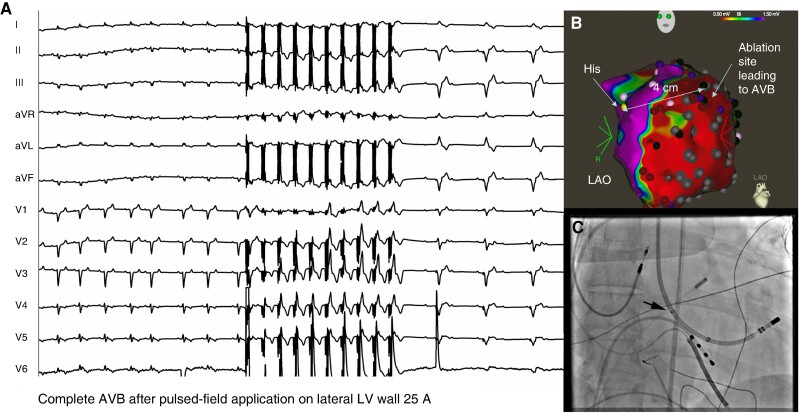
(*A*) shows the occurrence of complete AV block after PF ablation in a patient with non-ischaemic cardiomyopathy. (*B*) depicts an electroanatomical voltage map. The distance between the site of the application leading to the AV block and the location of His bundle recordings was 4 cm. (*C*) displays the fluoroscopic position of the ablation catheter. Note that the location of the proximal ring electrode on the catheter shaft is at the His bundle area (see text for further explanation). A, amper; AVB, AV block; His, his bundle recording site; LAO, left anterior oblique view; PF, pulsed field.

Transient conduction system block occurred in three cases (7%) during PF application on the lateral LV wall remotely from the conduction system. It consisted of complete AV block in one and left bundle branch block in two patients. Conduction blocks resolved in all cases within 1 h. Root analysis revealed that these events occurred during the retrograde approach to the LV. In such cases, the proximal shaft-visualizing electrodes of the ablation catheter were located close to the proximal portion of the conduction system at the LV aspect of the interventricular septum (*Figure 2*). Intracardiac echocardiography monitoring revealed that these unexpected adverse events were accompanied by the emission of microbubbles from these electrodes during PF energy delivery, suggesting the leakage of the current (see Supplementary material online, *Video S1*). Formation of the microbubbles could easily be prevented by covering the shaft electrodes with the sheath.

**Figure 3:**
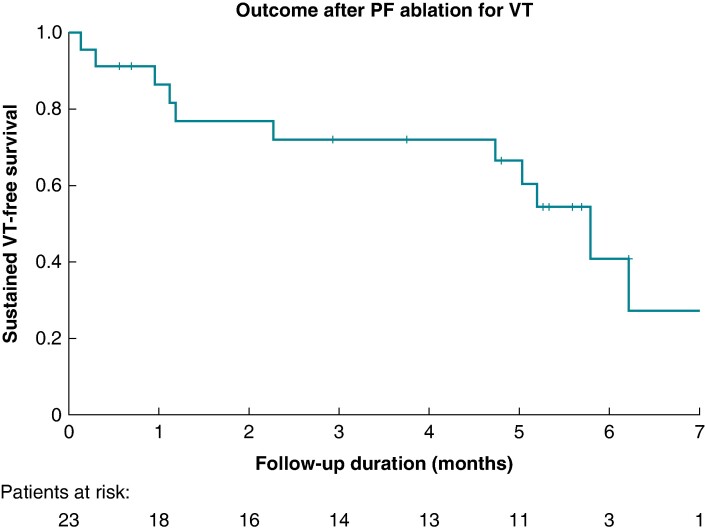
Kaplan–Meier curve of sustained VT-free survival after PF ablation. PF, pulsed field; VT, ventricular tachycardia.

Acute elimination of VPC was achieved in 17/21 (81%) patients and non-inducibility in 19/23 (83%) patients with VT. At the 3-month follow-up, persistent suppression of the VPCs was confirmed on Holter monitoring in 17/21 (81%) patients. The mean VPC burden decreased from 27 ± 12 to 7 ± 13% (reduction by 73 ± 51%, *P* < 0.001). In one patient with ectopy from posteromedial papillary muscle, acute suppression was achieved; however, the late recurrence of the same VPC morphology was observed at 3 months. On the other hand, in one patient who had acutely unsuccessful ablation, VPC disappeared during follow-up. In the group of patients with scar-related VT, the mean follow-up was 116 ± 75 days and 12/23 (52%) of patients remained free of any VT (*Figure 3*).

### Myocardial lesion size

Levels of hsTnT were assessed in 43% of patients before and after ablation and increased from 19 ± 12 to 600 ± 425 ng/L (*P* < 0.001). The increase was higher in patients with VT compared to those with VPC, but the difference was not significant (623 ± 446 ng/L vs. 323 ± 238 ng/L, *P* = 0.22).

## Discussion

The main findings of this study can be summarized as follows: (i) ablation of VA using PF delivered from a solid-tip 4 mm catheter is feasible with a high acute efficacy; (ii) despite favourable acute results, the recurrences are common in the VT group and acute non-inducibility may not be the optimal endpoint; (iii) compared to RF energy, focal PF ablation within the great cardiac vein was not limited by a high impedance or poor catheter-tip cooling and was not associated with coronary artery spasm; (iv) unexpected conduction system block was observed during retrograde CA in the LV due to current leakage from the proximal, shaft-visualizing electrodes of the ablation catheter; and (v) focal PF ablation was not associated with excessive myocardial damage as assessed by troponin levels post-ablation.

Compared to RF ablation of VA, PF energy offers several potential benefits. First, due to the non-thermal nature of PF, tissue overheating with a risk of steam pop is highly unlikely. Second, several preclinical studies suggested that PF can penetrate better into the scar tissue,^5,13,14^ which is particularly important in patients with scar-related VT. Third, PF applications are much shorter compared to RF and, thus, might be advantageous in some locations, where the stability of the catheter is challenging (e.g. on papillary muscle).^15^ This may also result in more favourable procedural times.

### Acute and short-term follow-up

While the focal PF ablation was quite successful (as assessed by acute suppression of the VPC or VT inducibility) in both groups, the short-term outcome in patients with VT was far less satisfactory. This may not be surprising, since the nature of VA is quite different in these patient cohorts. In the case of VPC, localized PF ablation has a higher chance of abolishing the focal source. On the other hand, the ablation target is far more extensive in scar-related VT, potentially also located more in-depth of the myocardial wall. In such a scenario, the studied PF energy delivery might not be effective enough and more pulses and/or higher energy deliveries were needed. However, this study reports one of the first larger experiences with PF ablation of VPC/VT that aimed at patients who failed RF ablation and safety was the primary interest. In addition, the pulse configuration used by the studied generator might not be ideal for VA ablation and could be further studied and optimized. Unfortunately, once PF ablation is delivered, local electrograms are instantaneously abolished and there is not much left, how to learn about the quality and durability of the created lesion. Finally, the explanation for the different efficacy of PF ablation in both groups might be a selection bias with more patients in the VT group having already previously unsuccessful RF ablation.

Regarding the assessment of the acute effect of PF ablation in scar-related VT, a new paradigm shift can be observed. In contrast to RF ablation, where the abolition of local abnormal electrograms was considered a reasonable endpoint of the substrate modification, PF delivery results in acute disappearance of the local electrograms, which may not reflect the creation of durable lesions. Acute lesions by PF compared to RF are known to have a much larger zone of reversible injury.^16^ This may also affect inducibility of VA at the end of the procedure. Thus, acute non-inducibility of VT after PF ablation might not be the optimal endpoint of the procedure. Whether the use of non-invasive programmed ventricular stimulation^17^ performed remotely from the ablation procedure could better assess the acute effect of ablation is to be investigated.

### Pulsed electric field delivery in the great cardiac vein

Application of RF energy in the great cardiac vein is often limited by the high impedance and temperature rise.^18^ Thus, alternative approaches, including alcohol venous injection,^19^ and bipolar ablation^20^ have been proposed. Pulsed electric field may pose another option for VPC originating in the LV summit, and experimental data have shown that PF is feasible in this scenario.^21^ Pulsed electric field ablation in the great cardiac vein has been also described in a clinical setting.^10^ Our current experience supports these observations. Based on clinical observations of coronary spasms, obtained with multielectrode PF delivery in the vicinity of the right coronary artery,^22^ the safety of PF ablation within the great cardiac vein is important. In this respect, we performed coronary angiography before and after PF delivery at a distance up to 2 mm (mean of 5 mm) to the coronary artery with no spasms noted. We can speculate that the lack of observed coronary spasms in our cohort could be due to the catheter design (4 mm tip vs. multispline catheter). Similarly to our experience, Brešković *et al.*^23^ have used a focal PF catheter within the coronary sinus for left-sided accessory pathways, and no clinically relevant spasms were reported. Nevertheless, our patient cohort was very small and the ablation catheter did not touch directly the coronary artery during any PF application. Thus, more data on the safety of this approach are still needed.

### Conduction system damage during pulsed electric field applications

The observations of transient conduction system blocks prompted us to evaluate the root cause of this phenomenon. Our explanation of these adverse events by leakage of the current through the proximal shaft-visualizing electrodes of the ablation catheter was confirmed by information obtained from the CENTAURI manufacturer. Because the high-voltage pulses are delivered to the tip of the ablation catheter during PF application, considerably high-voltage pulses are also synchronously delivered to proximal shaft-visualizing electrodes to prevent sparking and shortcutting between the wires within the catheter shaft. When these electrodes are in close proximity to the conduction system (such as during the retrograde access to the LV), the PF delivery may cause a transient conduction block. This explanation is supported by the preclinical studies that have described the high sensitivity of the conduction system to PF energy.^7^ Of note, this mechanism is specific only to the use of the SmartTouch ThermoCool™ catheter. The other catheters approved for the CENTAURI generator (i.e. TactiCath SE, Abbott and STABLEPOINT, Boston Scientific) do not have such electrodes on the shaft. But even for the SmartTouch catheter, the inadvertent damage of the conduction system could be prevented by covering and isolating these electrodes with the long sheath or by preferring the transseptal access to LV, which makes this adverse event unlikely.

### Myocardial damage

Pulsed electric field ablation leads to only moderate myocardial damage as assessed by troponin post-ablation increase. Studies assessing the troponin T dynamics in patients undergoing PF ablation of atrial fibrillation with a multielectrode catheter have reported much higher values (up to three times).^24,25^ Our observation is reassuring, since extensive myocardial damage in patients with scar-related VT and impaired LV ejection fraction may result in pump failure. On the other hand, PF may acutely affect a much larger area and this reversible zone of stunned ventricular myocardium may cause acute haemodynamic decompensation. Further studies are needed to clarify the haemodynamic risks associated with more extensive PF ablation in the ventricle.

### Study limitations

This was a prospective observational study aiming to describe the efficacy and safety of VA ablation by focal PF delivery in a spectrum of different VA. Thus, the small sample size may limit the validity of our observations, and additional studies with larger patient cohorts are needed to further explore the specific aspects and risks of focal PF ablation of VA in various patient populations. In addition, patients with arrhythmias in the vicinity of the proximal conduction system were on purpose not included in this study and no statement regarding safety/efficacy can be made in this respect. Finally, the observations made with the studied combination of the specific PF generator and ablation catheter cannot be extrapolated to other PF ablation technologies.

## Conclusions

Initial experience with the focal PF ablation of VA demonstrated high acute efficacy in ablation of both VPC and scar-related VT. However, the short-term success rate was more satisfactory in VPC patients, which reflects the size and complexity of the arrhythmogenic substrate and uncertainty about the endpoint of PF CA in scar-related VT. Pulsed electric field ablation was found particularly useful for ablation within the great cardiac vein.

## Supplementary Material

euae192_Supplementary_Data

## Data Availability

Data available upon request.
